# Load Transfer Theoretical Analysis of a Rigid Aircraft Pavement Contraction Joint Using a Novel Approach for Crack Characterization

**DOI:** 10.3390/ma19020376

**Published:** 2026-01-17

**Authors:** Sean Jamieson, Greg White

**Affiliations:** School of Science, Technology and Engineering, University of the Sunshine Coast, Sippy Downs, QLD 4556, Australia; sjamieson@usc.edu.au

**Keywords:** airport, load transfer, rigid pavement, aggregate interlock, finite element modeling

## Abstract

The contraction joints within paver runs are important for the design and construction of rigid aircraft pavements. These joints are typically un-doweled and sawn into the pavement to induce a crack. The joints control shrinkage cracking during curing, allow for thermal expansion and contraction, and provide load transfer through aggregate interlock joint stiffness between adjacent slabs. Aggregate interlock joint stiffness is typically modeled by assigning a spring element between two slabs that is indicative of the stiffness of the joint. However, that simplification may not accurately represent the complex interaction of irregularly shaped concrete faces and joint openings. Consequently, previous researchers have recommended modelling aggregate interlock stiffness based on physical crack shape. This research uses a novel approach to characterize crack shape through an idealized two-dimensional sinusoidal shape. Once the crack shape was defined, finite element methods were used to determine the significance of load, sublayer, and crack shape factors on load transfer values. It was determined that joint opening was the most significant factor for aggregate interlock load transfer. Future research is recommended to further validate the model against a larger data set, to confirm if the two-dimensional idealization of crack shape is an appropriate estimation of field conditions.

## 1. Introduction

Rigid aircraft pavements generally comprise unreinforced concrete slabs over a granular or bound sub-base layer, constructed on a natural, improved, or imported subgrade [1]. The pavement is divided into square or almost-square slabs to account for paver width and contraction joints. The contraction joints are located transverse to the paver runs and are typically un-doweled. These joints are sawn into the pavement at one-quarter to one-third depth to induce a crack, which controls shrinkage cracking during curing, allows for thermal expansion and contraction, and provides load transfer through vertical shear between adjacent slabs [2].

Load transfer is the ability of a joint in a pavement to transfer load from one slab to the next when trafficked. The edge stress is often the critical stress for pavement thickness design [3], and as a result, having effective load transfer reduces the required slab thickness. It is generally assumed that joints provide 25% load transfer to adjacent slabs [4]. However, load transfer is variable and affected by many factors, including loading regime, joint details, and sub-layer support [5]. For contraction joints, joint opening is reported to be the most important factor, with aggregate shape, size, strength, and particle distribution also significant due to their importance in determining crack shape [5,6,7,8]. Consequently, when assessing contraction joints for load transfer, a multi-factor analysis should be performed.

To assess load transfer, finite element (FE) methods have been used extensively [2]. FE methods solve mechanics problems by discretizing the problem domain into small elements, and then solving for a system of algebraic equations to determine physical properties such as displacements, stresses, and strains [9]. Earlier bespoke load transfer FE models were based on Westergaard theory, by assuming a two-dimensional medium-thick plate resting on a Winkler foundation, with load transfer modeled as a spring constant [10]. However, with the rapid advancements in computing technology, load transfer FE models now employ three-dimensional brick elements, using general-purpose FE programs, such as Abaqus [11]. Consequently, when assessing load transfer in rigid aircraft pavements with aggregate interlock joints, FE methods are well-suited.

Because earlier FE programs assumed a spring stiffness to model aggregate interlock interaction, the effect of joint opening and crack shape was simplified. Previous researchers have recommended physical crack shape be used to model load transfer [8], and with the advancement of texture measurement devices [12], crack shapes can now be accurately measured. Additionally, due to advanced contact interactions available in current commercial FE programs [13], the interaction between slabs can be modeled using physical crack shapes [14], in lieu of estimated spring constants. However, to balance computational efficiency with crack shape accuracy, shape simplifications are still required, such as approximating the crack profile shape to a sinusoidal wave.

This study used a novel approach to characterize the crack shape of a rigid aircraft pavement un-doweled contraction joint to be used in load transfer analysis. Firstly, the crack shape was idealized by a two-dimensional sinusoidal wave based on measurements of several concrete cores. The joint was then modeled in Abaqus CAE 2023, using a modified version of a previously validated load transfer model. Finally, a fractional factorial analysis and a focused, full factorial analysis were performed to assess the significance of load, sublayer, and joint factors on load transfer. Additionally, the relationships between typical load transfer values were investigated. This research aims to improve aggregate interlock load transfer modeling. Additionally, this research aims to inform designers and specifiers regarding the benefits and limitations of un-doweled aggregate interlock contraction joints, and their ability to provide load transfer under a variety of conditions.

## 2. Background

### 2.1. Rigid Aircraft Pavements

Rigid aircraft pavements typically comprise unreinforced 40 mm (maximum aggregate size) Portland cement concrete slabs over a sub-base layer, constructed on the subgrade [1]. The pavement is divided into square or almost-square slabs, typically ranging from 4 m to 8 m in length, to account for paver width and contraction joints. The contraction joints control shrinkage cracks during curing, allow for thermal expansion and contraction during daily and seasonal temperature cycles, and provide load transfer between adjacent slabs through aggregate interlock [2,15]. These joints are typically un-doweled, except for the last three rows of a pavement, in which case they may have dowels to prevent the joints from jacking open over time [4].

Initial design criteria for rigid aircraft pavements were developed around WWII and were based on Westergaard center-slab loading theory [16,17]. However, early verification tests in 1943 determined that stresses in edge loading were more severe [3]. Consequently, design criteria included a provision for either load transfer devices or the thickening of slab edges by 25%. It was assumed that properly designed joints would provide at least 25% load transfer to the adjacent slab [3], thus reducing the edge stress, and the load transfer assumption was included in subsequent design methods. The 25% load transfer value still remains in current pavement design systems, which have developed beyond Westergaard theory to now include layered elastic and FE methods, such as those used in the common aircraft pavement thickness determination tool FAARFIELD 2.1 [4,18,19].

### 2.2. Load Transfer

Load transfer is the ability of a joint in a concrete pavement to transfer load from one slab to the next when trafficked. Load transfer relies on joint stiffness, which is generally provided by stiffness from point devices, such as dowels, and stiffness from aggregate interlock [5]. However, for un-doweled contraction joints, joint stiffness is primarily provided by aggregate interlock [15]. Consequently, when joint openings are present due to periods of slab contraction, load transfer ability can be severely reduced [5,20].

For aircraft pavement applications, load transfer is generally characterized by load transfer efficiency via deflection (LTE_δ_) (Equation (1)) or the free-edge stress transferred (LT) (Equation (2)). LTE_δ_ can be relatively easily calculated from field measurements using a falling weight deflectometer (FWD) [21], with perfectly performing joints recording 100%. LT, however, cannot be as easily measured and is considered as how much stress is transferred to an unloaded slab, relative to the loaded slab, when under a free-edge loading condition. The free-edge condition is when a slab is loaded without an adjacent slab connected. This is the value typically used for design practices, has a range of 0% to 50%, and as discussed earlier, is assumed to be 25% in contemporary thickness determination methods.

(1) $${LTE}_{\delta }\;=\;\frac{\delta_{U}}{\delta_{L}}\;\times \;100$$

(2) $$LT\;=\;100\;\times \;\frac{\left(\varepsilon_{F}-\varepsilon_{L}\right)}{\varepsilon_{F}}$$

where

LTE_δ_ = deflection load transfer efficiency (%);
LT = percent of free-edge stress transferred (%);
δ_L_ = deflection of the loaded side of the joint (mm);
δ_U_ = deflection of unloaded side of the joint (mm);
ε_L_ = bending strain in the loaded slab edge at the joint;
ε_F_ = bending strain in the free-edge loading condition for the loaded slab edge at the joint.

### 2.3. Modeling Aggregate Interlock Load Transfer

Aggregate interlock is a shear load transfer mechanism and has historically been modeled as a set of linear springs, acting at each node alongside the joint discontinuity, with an assigned spring constant that is indicative of the stiffness of the joint [22]. This spring constant is a function of aggregate angularity, joint width, and aggregate hardness, but can also be affected by model geometry, material properties, and loading regime [6]. Although spring constants are a reasonable approximation for the effect of aggregate interlock joint stiffness on the global slab response, due to the many factors affecting the stiffness magnitude, assigning an appropriate stiffness value may be difficult, if not impossible, for a given scenario [6]. Consequently, there have been several developments to improve the joint stiffness calculation, and therefore the load transfer prediction, of un-doweled contraction joints.

Walraven [23] determined that both the maximum aggregate size and particle grading will affect the aggregate interlock crack shape and resultant shear stress. Consequently, they developed a model to represent crack shape in two-dimensional space with aggregate particles idealized as incompressible spheres of varying size, each intersecting the crack face at various depths depending on their statistical distribution within the concrete matrix. The shear and normal stresses at the crack result from the tangential and normal projections of the stresses produced when the mortar deforms plastically, as it bears on the aggregate particles. The shear stress and displacements for a given joint opening using the Walraven model were included in the bespoke load transfer FE program EverFE as a nonlinear shear transfer relationship [24].

Jensen [7] demonstrated that the crack shape followed the aggregate–mortar interface if the concrete mixture is made of hard aggregates [7], and developed a tri-linear spring stiffness relationship to represent joint stiffness. The relationship consisted of a period of initial slip, until the two crack faces were in contact, a sliding of the two crack faces, and finally a build-up of stresses in the crack. Jensen [7] further demonstrated that for small joint openings, the crack behavior was dominated by sliding, but for large joint openings, the initial free slip had more of an effect, and that concrete mixtures with larger stone sizes provide increased load transfer. Brink, Horak, and Visser [6] also found that stone size affected load transfer through laboratory and field testing, and developed a relationship to determine the relative movement of a loaded slab to an unloaded slab based on the joint opening and nominal maximum stone size. Maitra et al. [25] furthered that work to include this relationship as a spring stiffness value for determining LTE_δ_ in the FE program ANSYS.

Although the aforementioned developments have been significant in providing more accurate predictions of aggregate interlock load transfer, they generally rely on estimating slab interaction through a spring stiffness relationship and not the physical interaction between two irregularly shaped solid surfaces. Early work by Watter [8] acknowledged the benefits of measuring physical crack shape and relating that to a joint stiffness model. However, their work only determined the shape of the crack profile and did not relate it to load transfer. Furthermore, work by Vandenbossche [26] assessed crack profiles through volumetric measurements to assign a volumetric surface texture ratio (VSTR) to a crack face. Vandenbossche [26] determined that the VSTR of a crack affected load transfer, and provided a predictive equation that relates VSTR and crack width to LTE_δ_. Clearly, there is a relationship between the crack shape and load transfer ability of an aggregate interlock joint, and being able to model the physical crack shape within an FE model is of benefit. With advancements in FE technology, recent research has been able to model physical crack shape and measure stresses due to aggregate interlock interactions [14]. However, this is yet to be performed for aircraft pavement load transfer analysis.

There are multiple technologies, including laser profilometers and digital image analysis [8,12,27], that could be used to accurately measure crack shapes, which can then be drawn in FE programs. However, there will be a trade-off between the accuracy of the crack shape and the computational efficiency of each model run, requiring some form of shape simplification. Furthermore, using previously validated FE models can provide increased confidence in the accuracy of model outputs. For example, Jamieson and White [28] developed and validated a load transfer model for sinusoidal construction joints in Abaqus. The sinusoidal shape in that model could be altered to represent a measured crack profile of a real aircraft pavement, enabling detailed load transfer analysis to be performed. Consequently, this research idealized a measured crack shape into a two-dimensional sinusoidal shape for load transfer analysis, as discussed below.

## 3. Methods

**Figure 1:**
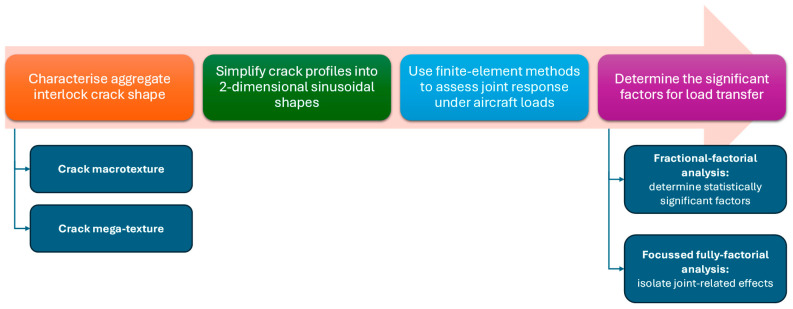
Methodology flow chart.

The objective of this research was to determine the relative effect that sublayer-, load-, and joint-related factors have on un-doweled contraction joint load transfer. In addition, the relationship between LT and LTE_δ_ was investigated. This was achieved by first characterizing the crack shape by an idealized two-dimensional sinusoidal shape. Secondly, an FE model was developed in Abaqus to simulate the idealized sinusoidal shape load transfer against a range of loading, sublayer, and joint conditions. Then a fractional factorial analysis was performed that included 21 model runs and a detailed statistical analysis. Finally, a focused, full factorial analysis was performed to isolate joint-related effects. Each of these steps is further described below, with a workflow summary provided in Figure 1.

### 3.1. Characterizing Aggregate Interlock

Two separate approaches to crack characterization were performed, one that used a laser texture meter to assess the profile shape of the crack macrotexture and one that assessed the crack mega-texture. Macrotexture is texture that has wavelengths between 0.5 mm and 50 mm, and mega-texture is texture that has wavelengths between 50 mm and 500 mm [29]. These two values are generally used to assess pavement skid resistance and rideability properties. However, this research aimed to see if either of these two scales could be used for crack characterization.

Four cores were extracted from existing aircraft pavements, two of which were sourced from a pavement that was older than 40 years, and the other two were sourced from a pavement that was approximately six years old. Both pavements were expected to be constructed to airport-quality concrete pavement specifications. However, the constituent materials were likely different due to the significant time between their construction, resulting in different cracking behavior. The older pavement cores are denoted as H1 and H2, with H representing relatively hard aggregate. That is because the crack profile went around the aggregates, indicating aggregates that were stronger than the concrete mortar. The newer pavement cores are denoted as S1 and S2, with S representing relatively softer aggregates. That is because the crack shape went through several aggregates, indicating that the mortar strength exceeded that of the aggregates.

**Figure 2:**
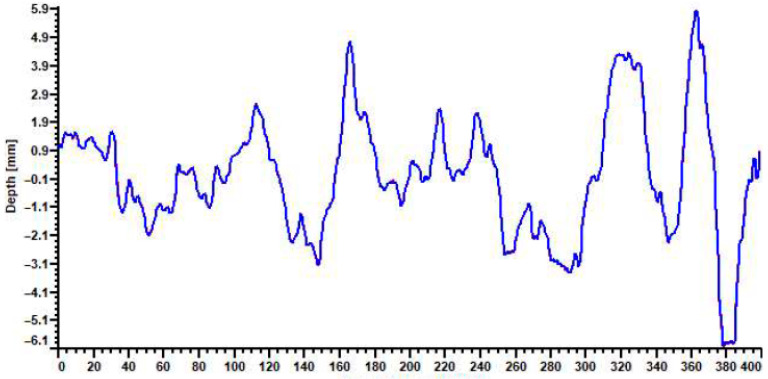
Measurement of MPD for H1 along the measured distance (mm for both axes).

To determine the macrotexture, an ELAtext laser texture meter, manufactured by IWS Messtechnik GmbH in Celle, Germany, was used. The ELAtext uses a rotating laser sensor that scans 400 mm of a surface with a horizontal resolution of 0.2 mm and produces a mean profile depth (MPD) [30]. The MPD is a two-dimensional representation of the difference between the arithmetic mean of the peak levels and horizontal baselines across a measured distance [31]. The laser texture meter was placed on the crack face, and profiles such as those presented in Figure 2 for an H1 crack face were produced. MPD recordings ranged from 3.01 mm to 3.08 mm for both H and S cores. Because of the small texture depth and lack of distinction between the H and S cores, it was determined that macrotexture shape was not a viable measurement for idealizing crack shape. That is because discretization would require extremely small elements, which would lead to very long computational run times. This also aligned with work by Chupanit [32], who determined weak-to-no correlation between traditional surface texture parameters and load transfer ability of an un-doweled contraction joint.

**Figure 3:**
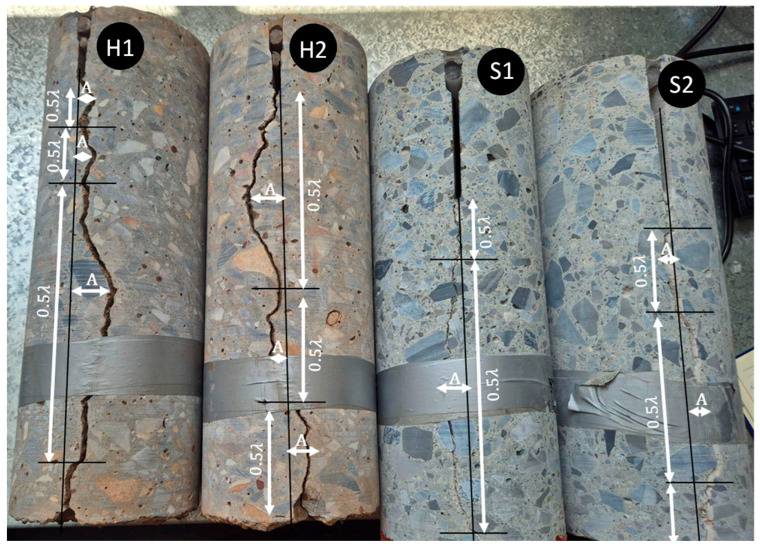
Measurement of crack profiles and idealization into sine wave characteristics.

The second approach assessed the mega-texture of the crack profile by simplifying changes in crack direction to half-wavelengths (0.5*λ*) and amplitudes (A), which would be averaged out to create a representative sinusoidal profile. As demonstrated in Figure 3, the difference in crack shape was clear for H cores compared to S cores, with H cores showing comparable A, but smaller 0.5*λ*, due to the crack profile following the aggregate edges. It is acknowledged that there is an element of subjectivity and approximation when determining A and 0.5*λ*, especially when considering that crack propagation is inherently non-uniform across three-dimensional space. However, this was necessary to ensure a simple, idealized sinusoidal shape for modeling. Furthermore, there was a considerable difference in the quantity, wave shape, and crack direction of each core side measured, owing to the non-uniform nature of crack formation. Consequently, the medians of all measurements were used, and hard aggregate joint faces were represented by an A of 10 mm and 0.5*λ* of 120 mm, and soft aggregate joint faces were represented by an A of 15 mm and 0.5*λ* of 200 mm. Additionally, further research is recommended to assess a larger data set of cores to determine whether the A and 0.5*λ* are similar for concretes of different ages and mixture properties.

### 3.2. Model Description

**Figure 4:**
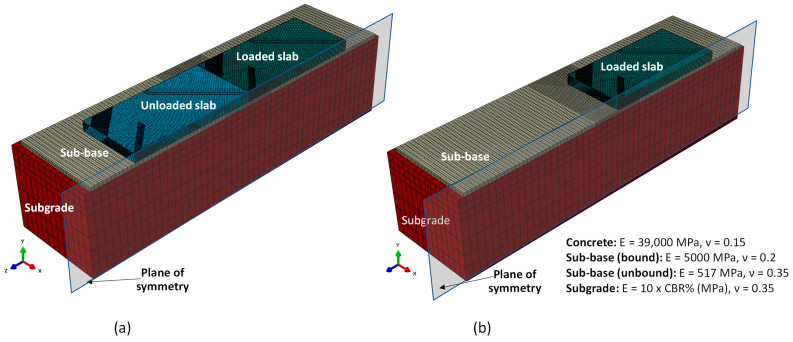
FE model, coordinate system, and material properties: (**a**) full model and (**b**) free-edge condition.

The FE model was built in Abaqus and comprised a 3 m deep subgrade under a 152 mm sub-base and two 350 mm thick concrete slabs, that were 4.6 m wide by 4.6 m long, connected by an un-doweled contraction joint. The joint was constructed in the XY-plane using the coordinate system and material properties defined in Figure 4, where E = elastic modulus; CBR = California Bearing Ratio; and ν = Poisson’s ratio. All materials were assumed to be elastic and isotropic, with no plastic deformation permitted. That is, joint face degradation was not considered in the model. Values for E and ν used typical values from current airport pavement thickness determination programs [4], except for concrete, which used a back-calculated modulus value from previous large-scale testing [11]. Because the model is symmetric along the X-axis, only half the model was required, and symmetrical boundary conditions were applied. To ensure appropriate calculation of the strain in the free-edge condition, the model was run in two conditions, one with the unloaded slab connected (Figure 4a) and another without the unloaded slab (Figure 4b).

Interface properties between each model instance were based on design assumptions for rigid aircraft pavements. A rough friction value with no separation was assigned to the subgrade-to-sub-base interface to simulate the no-slip condition of sublayers. Current rigid pavement design programs assume a frictionless surface between concrete slabs and sub-base; however, a partial bond will still exist [33]. Consequently, an almost frictionless Coulomb friction coefficient of 0.05 was applied, and separation was allowed to prevent the sub-base and concrete from behaving as a bonded surface. Finally, a Coulomb friction coefficient of 0.6 with allowable separation was used between the slabs to represent the interaction between two concrete faces [34]. This value was also used in the previous version of the FE model that assessed sinusoidal construction joints. However, further research is recommended to assess the effects of different Coulomb friction coefficients on load transfer response.

Boundary conditions included a fixed support at the bottom surface of the subgrade layer. Roller supports were applied to the subgrade and sub-base vertical faces to represent continuity of the material in the X-Z plane. The concrete slabs were allowed to move in the X-direction; however, the far edges were constrained in translation in the Y-axis and Z-axis, representing a constraint due to adjacent joints.

The model was meshed using eight-node linear brick elements with reduced integration and hourglass control (C3D8R) to minimize the effect of shear locking when subject to bending loads [35]. Advancing front sweep methods were used to mesh the sinusoidal aggregate interlock shapes at the concrete joint to ensure even distribution of elements around the curved features. An approximate vertical global mesh size of 22 mm was applied to the concrete slabs, which was demonstrated by an earlier mesh convergence study to provide acceptable results [11]. The sinusoidal shapes were assigned a finer mesh, with a minimum of 20 elements assigned per wavelength to ensure the curved shape was accurately represented. A coarser mesh was used for computational efficiency in areas of less interest, such as sub-layers and areas away from the joint.

**Figure 5:**
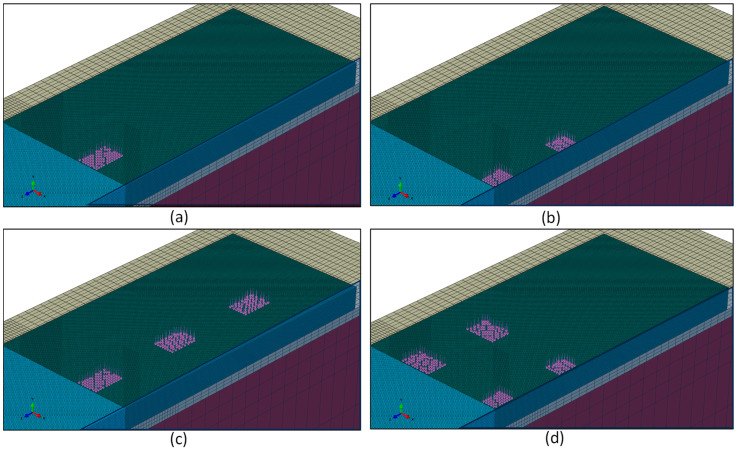
Aircraft loads across the plane of symmetry: (**a**) D-gear orthogonal, (**b**) D-gear parallel, (**c**) 3D-gear orthogonal, and (**d**) 3D-gear parallel.

Modern aircraft pavement thickness determination programs assume static loading, owing to static loads generally being more demanding than dynamic loads under normal operations [3,4]. Consequently, wheel loads were modeled as static rectangular pressure loads with tire pressure of 1.52 MPa and individual wheel load of 24.95 t, representing typical commercial aircraft tires. The Portland Cement Association technique for calculating pressure load size was used, resulting in a rectangular load area of 484 mm by 333 mm per wheel [36]. The wheel loads were applied in both dual (D-gear) and tridem (3D-gear) configurations across the plane of symmetry, in both orthogonal and parallel orientations to the edge of the joint, as shown in Figure 5. Furthermore, cyclic loading from repeated aircraft traffic was not considered. Consequently, this study only provides an initial joint response for a given load, joint, and sub-layer condition.

Model verification and validation are required to ensure that any new model represents observed physical events within the required tolerance and accuracy [37]. Verification is achieved through code verification and calculation verification. Because Abaqus is a widely used commercial software, there is high confidence in the code verification. However, calculation verification is still required to establish confidence that the selected discretization produces sufficiently accurate results [38]. This was achieved through a convergence study on the free-edge condition [11], which compared stress results against the bespoke load transfer FE program EverFE 2.24 [24]. For a D-gear with 22.68 t wheel loads, applied orthogonal to the joint at the edge of the slab, EverFE predicted a maximum concrete bending stress of 4.4 MPa. For the same loading condition, the model predicted 4.0 MPa concrete stress, or a 10% relative error, for element sizes of 30 mm. Reducing the element size further resulted in concrete stresses converging to 4.4 MPa. However, to ensure a suitable balance of computational effort and model accuracy, an approximate global mesh size of 22 mm was selected for the concrete slabs, which corresponded to a 5% relative error for concrete stress.

Previous versions of the model that assessed doweled construction joints and sinusoidal construction joints were validated against full-scale testing [28,39], which involved D-gear and 3D-gear wheel loadings ranging from 22.68 t to 31.75 t. The model over-predicted maximum strains by approximately 10%, but all load transfer model outputs were generally in the range of the full-scale testing. Although this model is largely based on the sinusoidal construction joint model, the idealization of the contraction joint shape to a sinusoidal shape has yet to be tested in the field. Consequently, further validation is required on un-doweled contraction joints, and until that time, this model remains purely theoretical. However, sound conclusions can still be drawn due to its similarity to the previous versions of the model.

### 3.3. Fractional Factorial Design

**Figure 6:**
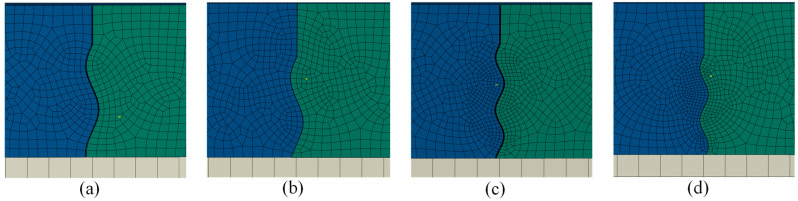
Joint shapes for modeling: (**a**) soft aggregate, quarter saw cut depth; (**b**) soft aggregate, third saw cut depth; (**c**) hard aggregate, quarter saw cut depth; (**d**) hard aggregate, third saw cut depth.

The fractional factorial experimental design assessed load-related factors, sublayer factors, and joint factors. Of the load-related factors, wheel configuration and wheel orientation were assessed, as previous research on construction joint load transfer demonstrated that these factors are statistically significant [40,41]. The D-gear wheel configuration represents an A320 aircraft, and the 3D-gear represents a B777 aircraft. To determine the significance of sublayer effects, subgrade strength and sub-base type were selected as factors. Of the joint-related factors, aggregate interlock shape, saw cut depth, and joint opening were included. Aggregate interlock shape was defined by concrete mixtures with relatively soft aggregates and concrete mixtures with relatively hard aggregates, using values for wavelength and amplitude, as previously discussed. Saw cut depth was either 90 mm or 120 mm, aligning with the extremes of typical practice, which allows for one-quarter- to one-third-depth cuts to induce a crack. The combination of aggregate quality and saw cut depth resulted in four different joint shapes, as shown in Figure 6.

Crack direction can have a significant effect on load transfer if the crack propagation skews from the plane perpendicular to the surface of the pavement [42], resulting in different load transfer results for approach and leave slabs for the same joint. However, due to the variable nature of crack propagation, as evidenced by the difference in crack shape on each side of the assessed cores (Figure 3), only crack direction perpendicular to the pavement surface was modeled. Additionally, like the Walraven model, the joint face was idealized in a two-dimensional space for computational efficiency. These are limitations of the model, and further research should also assess non-uniform crack skew across three dimensions to build upon the two-dimensional simplification in this research. The largest joint opening assessed was 3 mm, aligning with other studies that have demonstrated that the effect of joint opening on load transfer reaches an asymptote at approximately this magnitude [6].

To optimize the quantity of model runs, an orthogonal design was produced using specialized software known as SPSS Statistics V29 [43]. Orthogonality guarantees that the effect of one factor can be estimated separately from the effect of any other factor, thereby reducing the required number of model runs to produce significant results, compared to a full factorial analysis [44]. The factors and levels are described in Table 1, which resulted in 16 model runs. During testing, the high importance of joint opening became apparent; consequently, an additional five runs were performed using a joint opening of 2 mm. Therefore, a total of 21 model runs were performed.

### 3.4. Statistical Analysis

Upon the completion of model runs, a statistical analysis was performed using linear regression in the SPSS Statistics software [43]. This regression method was chosen due to its successful use on earlier versions of the FE model that assessed doweled and sinusoidal construction joints [28,40,41]. Output from a linear regression includes the R^2^ value, which indicates how much of the total variation in the dependent variables can be explained by the experimental factors, and the analysis of variance (ANOVA) significance, which indicates the statistical significance of the regression model. Where the *p*-value is below 5%, the regression model statistically significantly predicts the outcome variable [45].

On each linear regression, each experimental factor is assigned a non-standardized coefficient (B), which can be used in a predictive linear equation; a standardized coefficient (β), which ranks the relative importance of the relative effect of each factor on the dependent variable; and a *p*-value, which indicates the significance of each factor. The initial linear model included all experimental factors, as well as interactions for joint opening with all other experimental factors. The joint opening interaction was included because it was expected that some experimental factors would become significant only when there was an opening present. For each dependent variable (LT and LTE_δ_), multiple regressions were then performed, iteratively removing the least significant factor (with the highest *p*-value) until only significant independent factors remained (*p*-values < 0.05), following common statistical analysis methods [46]. Therefore, the final linear regression model only included experimental factors and interactions that were statistically significant.

### 3.5. Focused, Full Factorial Design

Due to evidence of complex interactions during the fractional factorial analysis and several zero-load transfer results, a focused, full factorial analysis was also performed that isolated the effects of joint opening, crack shape, and saw cut depth for a defined loading and subgrade condition. The defined condition was a 3D-gear parallel load on a bound sub-base over a subgrade with CBR 3%. The heavy loads and soft subgrade were chosen to increase the likelihood of non-zero results. The same factors for saw cut depth and aggregate quality from Table 1 were used, and joint openings were modeled as 0 mm, 1 mm, 2 mm, and 3 mm. In total, there were 16 model runs performed for the focused, full factorial analysis.

## 4. Fractional Factorial Analysis Results and Discussion

### 4.1. Results

**Figure 7:**
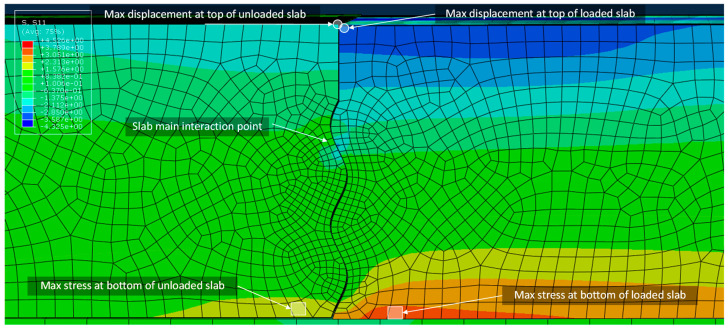
Typical model results showing elements used for load transfer calculations.

Typical model output and the elements used for load transfer calculations are shown in Figure 7. Results for the fractional factorial experimental design are presented in Table 2, and linear regression findings are presented in Table 3. Values for calculation of LTE_δ_ and LT were identified as the elements at the bottom of the slabs with the highest tensile stress (and corresponding strain) along the X-direction, aligning with design assumptions for rigid pavements [4]. For 3D-gears in a parallel orientation, the loaded slab elements selected were directly below the central wheel load closest to the joint. The same plane was used for D-gears in parallel orientations. For 3D-gears and D-gears in an orthogonal orientation, the elements selected were directly below the wheel load closest to the joint. Elements used for the unloaded slab were those with the maximum values along the same plane as the elements used for the loaded slab. Values for LTE_δ_ were selected as the nodes at the top of the slabs that experienced the largest vertical deflection. For all load combinations, these values aligned with the nodes that intersected the joint edge and the plane of symmetry. LTE_δ_ and LT results recorded as zero indicate no interaction between the loaded and unloaded slabs.

Of interest is that the interaction between the two slabs only occurred at a single point, which was located between 0.25*λ* and 0.5*λ* below the saw cut for all joint arrangements, as shown in Figure 7. This interaction was similar to previous research on sinusoidal joints that determined the uppermost sine wave is the main point of interaction between two concrete slabs [28]. The stresses at this point were generally non-uniform, and in some cases, the stresses exceeded the tensile strength of typical concrete mixtures, owing to the complex contact stresses caused by the sliding of the unloaded and loaded slabs.

### 4.2. Discussion

Joint opening and joint opening interactions were determined to have the greatest effect on LT, evidenced by their statistical significance (*p* < 0.05) and large β-values. This aligns with previous full-scale testing, which determined that joint opening was the single most important factor for un-doweled contraction joints [47]. Wheel configuration, sub-base type, aggregate quality, and saw cut depth were also statistically significant, but only when combined with a joint opening. Subgrade strength was significant for LT without a joint opening present, with weaker subgrades providing increased LT. Decreasing subgrade strength by a 1% CBR raised the LT value by an equal value due to allowing the loaded slab to deflect more, and therefore engage more with the unloaded slab. This CBR-to-LT relationship has also been observed in doweled joints and sinusoidal joints using the same FE model [28,40,41]. Joint opening was the only factor found to be statistically significant for LTE_δ_, further highlighting the importance of this factor for un-doweled contraction joints.

**Figure 8:**
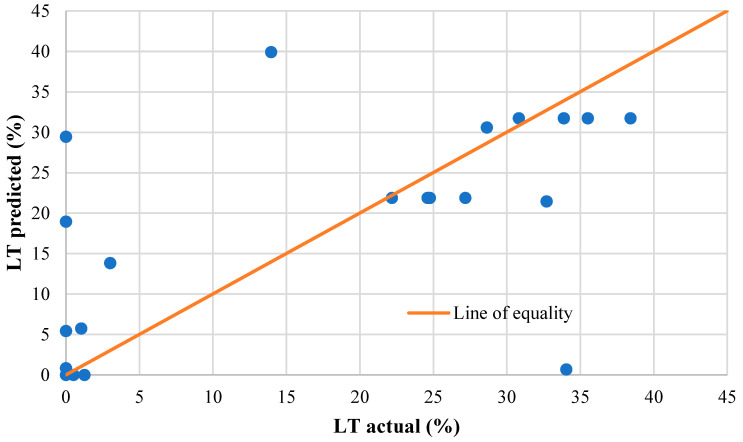
Actual LT from model runs versus predicted LT values using linear regression.

The linear regression for LT achieved a relatively high R^2^ value of 0.83 and a *p*-value of < 0.05, demonstrating a statistically significant relationship with high correlation. Similarly, LTE_δ_ achieved a high R^2^ value of 0.71 and produced a statistically significant relationship. However, due to the very large standard error in both LT and LTE_δ,_ it was expected that the high correlation was due to the large number of zero values. To test this, predictive LT values were graphed against actual LT values from the FE model, as shown in Figure 8. The predictive values used the B-values from Table 3 as coefficients for the factors and factor interactions in a linear equation. If the predictive LT was accurate, all values should sit on the line of equality. However, it is obvious that the linear relationship has significant errors and will over-predict LT, especially where the actual LT is recorded as zero. Consequently, the linear regression is not suitable as a predictive equation for the LT of aggregate interlock joints. Therefore, further analysis was performed that isolated the effects of joint opening, crack shape, and saw cut depth, as discussed earlier, with results presented below.

## 5. Focused, Full Factorial Analysis Results and Discussion

### 5.1. Results

**Figure 9:**
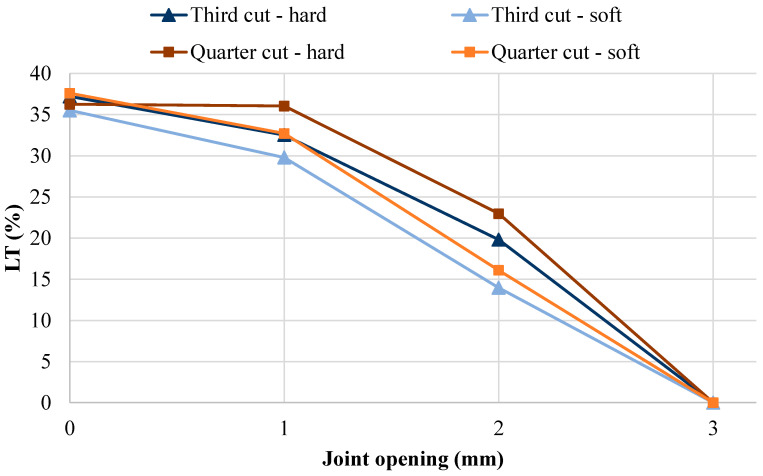
LT for four joint shapes versus joint opening.

**Figure 10:**
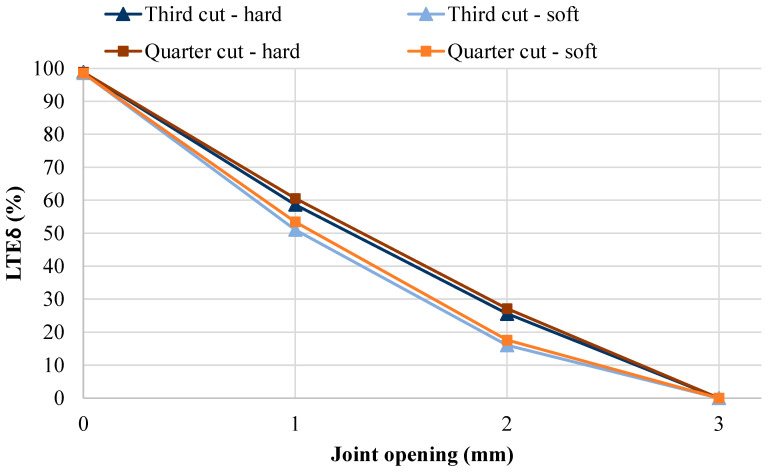
LTE_δ_ for four joint shapes versus joint opening.

Table 4 shows the results for the focused, full factorial analysis, where wheel configuration, wheel alignment, sub-base type, and subgrade CBR were kept constant. Figure 9 and Figure 10 provide plots of the four joint shapes analyzed versus the joint opening, for LT and LTE_δ_, respectively.

### 5.2. Discussion

As demonstrated in Figure 9, the effect of joint opening is more substantial on LT than saw cut depth or aggregate quality. For example, the mean difference in LT for a 1 mm, 2 mm, and 3 mm joint opening, compared to a no-gap condition, was 3.3%, 19.2%, and 35.4%, respectively, with the zero-load transfer condition estimated between 2 mm and 3 mm, and confirmed at 3 mm. These results compare well with laboratory testing by Brink, Horak, and Visser [6], who determined that load transfer reaches an asymptote at 2.5 mm and 4 mm joint openings for 19 mm and 37.5 mm maximum aggregate size mixtures, respectively. The results also compare well with research by Colley and Humphrey [15], who determined an absence of load transfer effectiveness at joint openings of 2 mm and 4.5 mm in laboratory and field testing, respectively. The FE model prediction of zero load transfer between 2 mm and 3 mm joint opening is at the lower end of the previously mentioned field and laboratory observations. However, this can be attributed to simplifications in the FE model, and the fact that aircraft slabs (and the slab in this model) were significantly thicker than those assessed by Colley and Humphrey [15] and Brink, Horak, and Visser [6], who investigated road pavements, which are relatively thinner. Because flexural rigidity is increased for the thicker slabs [5], less movement is enabled, making it more difficult for the loaded slab to interact with the unloaded slab as the joint opening increases, when compared to road pavements.

The change in LT due to aggregate interlock shape had a mean of 2.4% for all joint opening conditions and a maximum difference of 6.9% for a joint opening of 2 mm, with harder aggregate joints generally achieving higher LT. This was due to the smaller wavelength and, therefore, more horizontal interaction between the slabs for the harder aggregate joints compared to softer aggregate joints. The change in saw cut depth had less effect, with a mean difference of 1.6% LT across all joint opening conditions, and a maximum difference of 3.5% for a joint opening of 1 mm, with quarter-depth saw cuts providing greater LT than third-depth saw cuts. This may suggest that practice should always use quarter-depth saw cuts to maximize LT. However, as discussed earlier, the main interaction between the two slabs occurs at the top 0.25*λ* to 0.5*λ* of the idealized joint face. Because this location is dependent on both the controlled saw cut depth and random, induced aggregate interlock crack shape, it is expected that there will be many scenarios where third-depth cut joints will achieve higher LT than quarter-depth cut joints. Consequently, the focus of the saw cut depth should remain the successful induction of a crack, and should not focus on maximizing LT.

**Figure 11:**
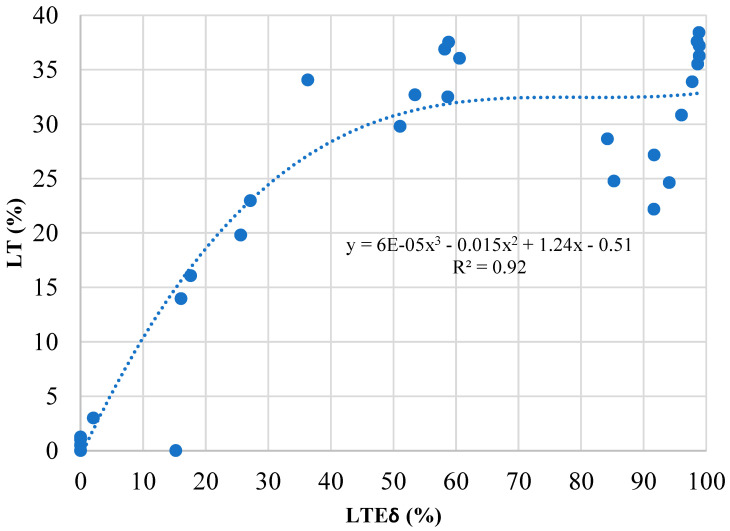
LTE_δ_ versus LT for an aggregate interlock joint.

Another observation from Figure 9 and Figure 10 is that the rate of LT decline is not consistent for both LT and LTE_δ_; that is, going from a closed joint to a 1 mm joint opening had a minor effect on LT, but moving to a 2 mm joint opening had a much larger effect. Conversely, the rate of LTE_δ_ decline is almost linear across the joint openings analyzed. Because LTE_δ_ can be calculated in the field using FWD measurements, Figure 11 was developed to aid practitioners in determining LT for aggregate interlock joints based on calculated LTE_δ_ values, using all results from this research. The relationship between LTE_δ_ and LT was best determined to be represented by a cubic equation. Although a relatively high R^2^ value was recorded (0.92), it is noted that there is still significant data dispersion, especially for LTE_δ_ values greater than 30%. Consequently, the cubic relationship can only be used as an approximation. This is different from the findings of previous research on doweled joints and sinusoidal joints, which determined that the relationship between LTE_δ_ and LT could be represented by non-linear power equations [48]. However, in that research, it was determined that the relationship of LTE_δ_ to LT is dependent on joint type. Consequently, Figure 11 can be used for aggregate interlock joints, but is not applicable to sinusoidal and doweled joints. Additionally, to achieve the LT design assumption of 25%, LTE_δ_ calculations should achieve or exceed 30%.

## 6. Conclusions

Based on 21 model runs using a fractional factorial analysis, it was concluded that joint opening is the most important factor for aggregate interlock joint load transfer. However, wheel configuration, sub-base type, crack shape, and saw cut depth were also significant when the joint was open. Furthermore, subgrade strength also affected LT, regardless of the joint opening width, with weaker subgrades providing greater LT. On completion of a further 16 model runs using a focused, full factorial design, it was concluded that LT reduces to zero between 2 mm and 3 mm joint openings, and the combination of saw cut depth and crack shape will have an effect on LT values. However, this effect was not as pronounced as that of joint opening. Finally, based on all model runs, it was concluded that the relationship between LT and LTE_δ_ can be represented by a cubic equation, and this equation could be used as a prediction for design-based LT when using LTE_δ_ values calculated from in-field FWD measurements. However, a limitation of this research is that it is purely theoretical, and additional research is recommended to further validate the model and to confirm if the two-dimensional idealization of crack shape is an appropriate estimation of field conditions, using a larger data set of concrete cores.

## Figures and Tables

**Table 1:** Factors and levels for orthogonal design.

Factor	Level A	Level B	Level C
Wheel configuration	D	3D	-
Wheel orientation	Orthogonal	Parallel	-
Subgrade CBR	3%	13%	-
Sub-base type	Unbound	Bound	-
Aggregate quality	Hard aggregate	Soft aggregate	-
Saw cut depth	¼ slab (90 mm)	⅓ slab (120 mm)	-
Joint opening	0 mm	1 mm	3 mm

**Table 2:** Fractional factorial model results (O = orthogonal; P = parallel; U = unbound; B = bound).

Run No.	Wheel Configuration	Wheel Orientation	Sub-Base Type	Subgrade CBR (%)	Aggregate Quality	Saw Cut Depth	Joint Opening (mm)	LTE_δ_ (%)	LT (%)
1	3D	O	U	3	H	1/3	1	84.2	28.6
2	3D	O	B	13	H	1/4	1	0	0
3	3D	P	U	3	H	1/4	0	98.8	38.4
4	D	O	U	13	S	1/4	0	85.3	24.8
5	D	O	B	3	S	1/3	0	96.0	30.8
6	3D	O	U	13	S	1/3	3	0	0
7	D	P	B	13	H	1/4	3	0	0
8	3D	P	U	13	S	1/4	0	91.7	27.2
9	3D	P	B	13	H	1/3	0	94.1	24.6
10	D	O	U	3	H	1/4	0	97.8	33.9
11	D	O	B	13	H	1/3	0	91.6	22.2
12	D	P	B	3	S	1/4	1	2.03	3.02
13	D	P	U	13	S	1/3	1	0	0
14	3D	O	B	3	S	1/4	3	0	0
15	D	P	U	3	H	1/3	3	0	0
16	3D	P	B	3	S	1/3	0	98.6	35.5
17	3D	O	U	3	H	1/3	2	15.2	2.6
18	3D	P	U	3	H	1/4	2	36.3	34.1
19	3D	P	B	3	S	1/3	2	16.0	13.9
20	D	P	U	3	S	1/3	2	0	0
21	3D	P	B	3	S	1/4	1	53.4	32.7

**Table 3:** Linear regression output for load transfer characterization (‘*’ indicates an interaction of two factors).

LT (%)				
Factor	B	Standard Error	β	Significance
Constant	34.7	3.5	-	<0.001
Subgrade	−1.0	0.3	−0.32	0.012
Joint opening	−30.5	5.2	−2.33	<0.001
Joint opening * wheel configuration	7.6	2.4	0.53	0.007
Joint opening * sub-base type	12.6	3.9	0.85	0.006
Joint opening * aggregate quality	7.4	2.5	0.50	0.009
Joint opening * saw cut-depth	14.4	4.1	0.97	0.003
*Regression summary: R^2^ = 0.83; standard error = 7.5; ANOVA significance ≤ 0.001*				
**LTE_δ_ (%)**				
**Factor**	**B**	**Standard error**	**β**	**Significance**
Constant	82.2	7.6	-	<0.001
Joint opening	−31.2	4.6	−0.84	<0.001
*Regression summary: R^2^ = 0.71; standard error = 24.5; ANOVA significance ≤ 0.001*				

**Table 4:** Full factorial experimental results.

Run No.	Wheel Configuration	Wheel Alignment	Sub-Base Type	Subgrade CBR (%)	Aggregate Quality	Saw Cut Depth	Joint Opening (mm)	LTE_δ_ (%)	LT (%)
1	3D	P	B	3	H	1/3	0	98.9	37.2
2	3D	P	B	3	S	1/3	0	98.6	35.5
3	3D	P	B	3	H	1/4	0	98.9	36.3
4	3D	P	B	3	S	1/4	0	98.5	37.6
5	3D	P	B	3	H	1/3	1	58.7	32.5
6	3D	P	B	3	S	1/3	1	51.1	29.8
7	3D	P	B	3	H	1/4	1	60.6	36.1
8	3D	P	B	3	S	1/4	1	53.4	32.7
9	3D	P	B	3	H	1/3	2	25.6	19.8
10	3D	P	B	3	S	1/3	2	16.0	14.0
11	3D	P	B	3	H	1/4	2	27.1	23.0
12	3D	P	B	3	S	1/4	2	17.6	16.1
13	3D	P	B	3	H	1/3	3	0	0
14	3D	P	B	3	S	1/3	3	0	0
15	3D	P	B	3	H	1/4	3	0	0
16	3D	P	B	3	S	1/4	3	0	0

## Data Availability

The original contributions presented in this study are included in the article. Further inquiries can be directed to the corresponding author.
